# Speed, energy and area optimized early output quasi-delay-insensitive array multipliers

**DOI:** 10.1371/journal.pone.0228343

**Published:** 2020-02-03

**Authors:** P. Balasubramanian, D. L. Maskell, N. E. Mastorakis

**Affiliations:** 1 School of Computer Science and Engineering, Nanyang Technological University, Singapore; 2 Department of Industrial Engineering, Technical University of Sofia, Sofia, Bulgaria; University of Glasgow, UNITED KINGDOM

## Abstract

Multiplication is a widely used arithmetic operation that is frequently encountered in micro-processing and digital signal processing. Multiplication is implemented using a multiplier, and recently, QDI asynchronous array multipliers were presented in the literature utilizing delay-insensitive double-rail data encoding and four-phase return-to-zero (RTZ) handshaking and four-phase return-to-one (RTO) handshaking. In this context, this article makes two contributions: (i) the design of a new asynchronous partial product generator, and (ii) the design of a new asynchronous half adder. We analyze the usefulness of the proposed partial product generator and the proposed half adder to efficiently realize QDI array multipliers. When the new partial product generator and half adder are used along with our indicating full adder, significant reductions are achieved in the design metrics compared to the optimum QDI array multiplier reported in the literature. The cycle time is reduced by 17%, the area is reduced by 16.1%, the power is reduced by 15.3%, and the product of power and cycle time is reduced by 29.6% with respect to RTZ handshaking. On the other hand, the cycle time is reduced by 13%, the area is reduced by 16.1%, the power is reduced by 15.2%, and the product of power and cycle time is reduced by 26.1% with respect to RTO handshaking. Further, the RTO handshaking is found to be preferable to RTZ handshaking to achieve slightly improved optimizations in the design metrics. The QDI array multipliers were realized using a 32/28nm complementary metal oxide semiconductor (CMOS) process technology.

## 1. Introduction

Multiplication is a fundamental arithmetic operation that is frequented in micro-processing and digital signal processing. Multiplication is implemented using a multiplier, and the multiplier can be implemented in synchronous and asynchronous design styles. Many synchronous multipliers exist [1], and some non-robust [2–12], and few robust asynchronous multiplier designs [13–16] have been reported in the literature. References [2–12] discuss different asynchronous multiplier designs, which are either full-custom or semi-custom designs and make use of a non-robust, non-delay insensitive two-phase bundled-data asynchronous handshake protocol for data processing and communication. Although bundled-data asynchronous multipliers are likely to be better off than the synchronous multipliers due to the formers’ ability to achieve average-case speed performance and low power compared to the worst-case speed performance of the latter, they are not robust. This is because bundled-data asynchronous multipliers would incorporate a matched-delay element and the worst-case delay of this element is configured to match the worst-case delay of the multiplier logic and the timing overhead to accommodate the signal set-up and hold times incurred during two-phase handshaking. The two-phase asynchronous handshaking requires the explicit exchange of request and acknowledge signals between the input and output registers of an asynchronous circuit stage. Supposing due to a parametric or process or temperature variation, if the worst-case delay of the multiplier logic may exceed the delay of the matched-delay element, new input data may be allowed to be supplied to the bundled-data asynchronous multiplier before the processing of the current input data is completed, which may give rise to erroneous data processing violating the handshaking. Therefore, bundled-data asynchronous multipliers are not delay-insensitive (DI).

On the other hand, the QDI asynchronous design style [17], proposed as a self-timed design approach in [18], is a robust, reliable and practically realizable delay-insensitive design style suitable for micro- and nano-electronics [19]. QDI circuits have been shown to be Turing-complete [20], are correct-by-construction, and robust since they possess the inherent ability to cope with process, parameter and temperature variations [21–23]. A notable feature of QDI circuits is that they are able to wait for the arrival of all the inputs and would allow the passage of new inputs only after the current inputs are processed and acknowledged to the environment.

References [13–16] discuss QDI array multipliers assuming an example 4×4 multiplication. Kim *et al*. [13] compared 2-dimension pipelining versus 1-dimension pipelining for a QDI array multiplier realized using custom-designed null convention logic (NCL) gates. It was concluded that the former pipelining approach leads to an improved speed than the latter albeit at the expense of increases in area and power. Metku *et al*. [14] also custom-realized 4×4 QDI array multipliers using NCL gates which were designed using static CMOS and gate diffusion input (GDI) [24,25] techniques. They observed that the GDI design style results in reduced area and less average power compared to the static CMOS implementation. However, [13] and [14] involve manual (i.e., full-custom) transistor-level designs for each gate to realize the multipliers and they do not make use of a semi-custom design flow i.e., a standard digital cell library for synthesis.

On the other hand, [15] and [16] present QDI array multipliers realized using a standard digital cell library [26]. The Muller C-element [27] is alone custom-realized and included as a part of the digital gate library for implementing the QDI multipliers. The C-element is basically a rendezvous element that would wait for the arrival of all its inputs. If all the inputs to a C-element are binary 1 or 0, its output would be 1 or 0 respectively. If all the inputs are not the same, the C-element would retain its existing steady-state. Compared to [15], [16] presented a better asynchronous partial product generator that led to some optimizations in design metrics for the QDI array multipliers. However, [15] and [16] realized QDI array multipliers only using QDI full adders pertaining to different self-timed design methods [28–33]. In this work, we propose an improved asynchronous partial product generator and a new asynchronous half adder which are used in conjunction with the full adder of [33] to realize speed, energy and area efficient QDI multipliers.

Contrary to the notion of DI circuits, which are infeasible, QDI circuits are practically realizable. This is because the C-element and the inverter (and the non-inverting buffer) are alone DI and the rest of the logic gates (both simple and complex) are not DI. A gate is said to be DI if a change of its output to either of the two binary states unambiguously indicates the state of its inputs after the application of new input data. For examples, when the output of a C-element changes from 1 to 0 or 0 to 1, it implies that all its inputs have changed from 1 to 0 or 0 to 1 respectively, and if the output of an inverter changes from 1 to 0 or 0 to 1, it implies that its input has changed from 0 to 1 or 1 to 0 respectively. Hence, the outputs of the C-element and the inverter are said to indicate i.e., acknowledge the state of their inputs unambiguously.

However, practically, a digital logic circuit cannot be constructed using just the C-element and the inverter. This necessitated the introduction and assumption of isochronic forks [17] as the weakest possible compromise to delay-insensitivity. An isochronic fork basically refers to a signal node/junction with more than one wire branching out from that node or junction. If a rising or a falling signal transition occurs at an isochronic node, all the wires forking out from that node are assumed to experience similar signal transitions occurring concurrently. The isochronic fork assumption enables the practical realization of DI circuits, which are called QDI circuits.

Given the phenomenon of variability, which has assumed significance in the nanoelectronics regime, robust asynchronous designs of multipliers pertaining to the QDI design style is important. Although many high-speed multiplier architectures exist, the array multiplier is a good choice especially for low power and low frequency applications such as a hearing aid [9]. Moreover, the array multiplier architecture is regular and convenient to layout than the other multiplier architectures [1]. A speed, energy and area optimized QDI array multiplier is presented in this work.

The rest of this article is organized into four sections. Section 2 discusses the fundamentals of QDI circuit design. Section 3 describes a QDI array multiplier which incorporates the proposed partial product generator and the half adder. The problem with realizing a QDI array multiplier using only early output building blocks such as half adders and full adders and partial product generators is also discussed, and a solution for the same is given. Section 4 provides the implementation results for various QDI array multipliers pertaining to RTZ and RTO handshaking. Finally, we conclude in Section 5.

## 2. Fundamentals of QDI circuit design

**Fig 1:**
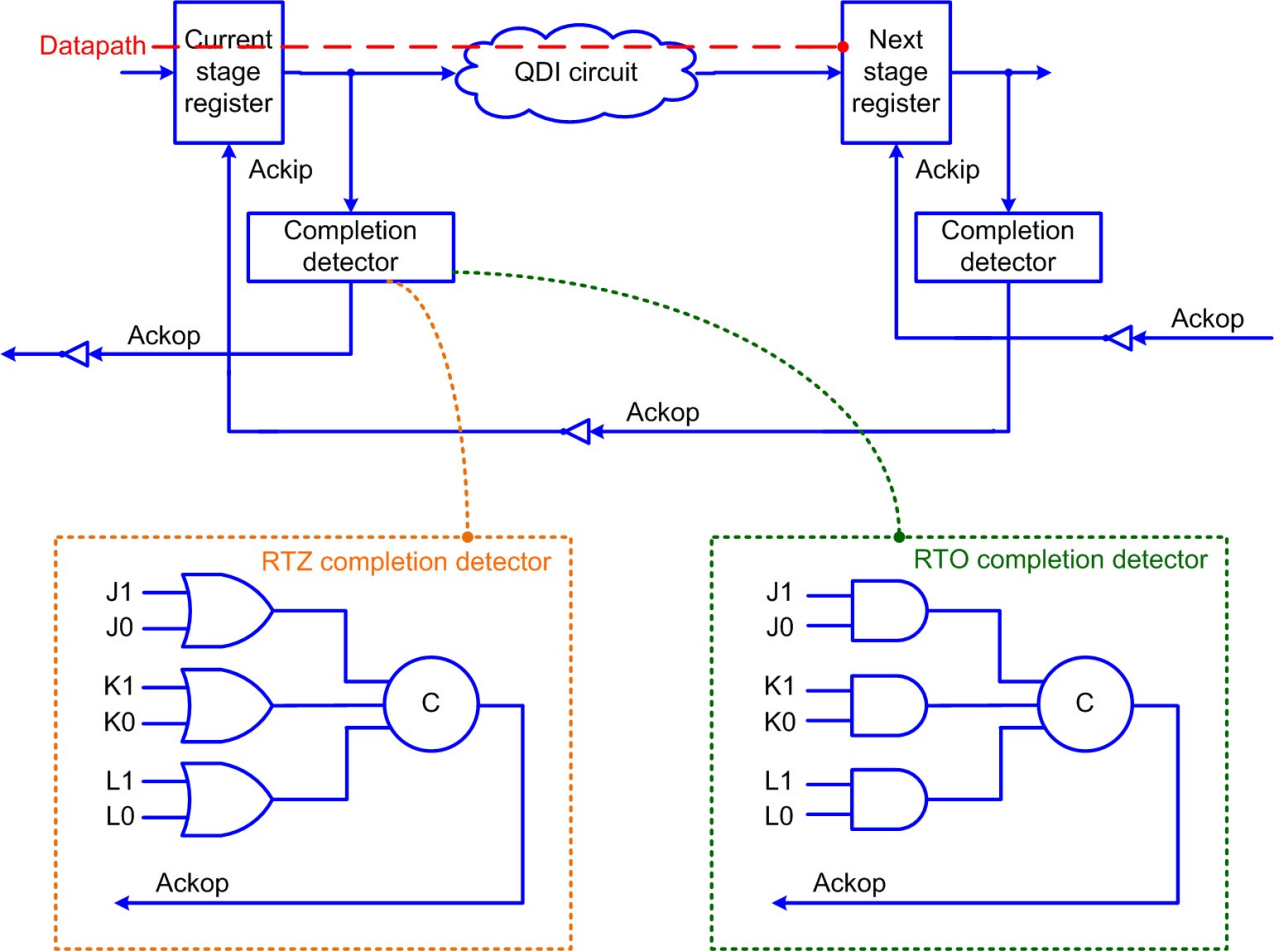
Block diagram of a QDI circuit stage. The QDI circuit is sandwiched between the current stage and next stage registers. Ackip and Ackop are ‘acknowledge input’ and ‘acknowledge output’ signals, which are complementary to each other. The critical data path is shown by the dashed red line which traverses a current stage register and the QDI circuit. Completion detectors for example double-rail inputs (J1, J0), (K1, K0) and (L1, L0) pertaining to RTZ and RTO handshaking are shown within the dotted orange and green boxes.

A background about QDI circuit design is given in this section to familiarize the reader. Fig 1 shows the block schematic of a QDI circuit stage. The QDI circuit is sandwiched between the current stage and next stage registers. A register is allotted for each of the rails of a double-rail encoded primary input, and the register is a 2-input C-element. In the figures, the circles having the marking ‘C’ represent the C-elements.

### 2.1. Double-rail data encoding and four-phase RTZ handshaking

In Fig 1, (J1, J0), (K1, K0) and (L1, L0) represent the double-rail encoded primary inputs of the single-rail inputs J, K and L. According to RTZ handshaking, the double-rail data encoding is defined as shown in Table 1 by considering an example single rail input W. In Table 1, W1 and W0 represent the two encoded rails of W. In general, W1 is called the true rail and W0 is called the false rail of a double-rail encoded single rail input W. W = 0 is specified by W0 = 1 and W1 = 0, and W = 1 is specified by W1 = 1 and W0 = 0. W1 = W0 = 0 is the (all-zeroes) spacer and W1 = W0 = 1 is termed illegal (i.e., indeterminate) since the coding scheme must be complete [34] and unordered [35] to ensure the delay-insensitivity.

**Table 1:** Double-rail data encoding following RTZ handshaking.

Single-rail input	Double-rail encoded input		State Interpretation
W	W1	W0	
0	0	1	Binary 0
1	1	0	Binary 1
–	0	0	Spacer
–	1	1	Indeterminate

The application of inputs to a QDI circuit adhering to the four-phase RTZ handshaking follows the sequence of *data-spacer-data-spacer* and so forth. The data and spacer are supplied alternately implying that an RTZ phase occurs between successive applications of data. The RTZ phase paves the way for a robust data communication (i.e., handshaking) between the current stage and next stage registers.

The four-phase RTZ handshaking involves four steps. First, the double-rail data bus initially assumes the spacer and the acknowledge input, Ackip = 1. After the current stage register transmits a data, rising signal transitions i.e., binary 0 to 1 would occur on anyone of the rails of the double-rail data bus. Second, the next stage register would receive the processed data and would drive the acknowledge output (Ackop) to 1. Third, the current stage register would wait for Ackip to assume 0 and after this occurs, the double-rail data bus would be reset, i.e., the double-rail data bus would assume the spacer again. Fourth, after a finite and positive unbounded time duration elapses, the next stage register would drive Ackop to 0 and Ackip would assume 1. With this, one data transaction is said to be completed and the QDI circuit is allowed to resume the next data transaction.

### 2.2 Double-rail data encoding and four-phase RTO handshaking

According to RTO handshaking [36], the double-rail data encoding is defined as shown in Table 2 by considering an example single rail input W, with W1 and W0 representing the two encoded rails of W. W = 0 is specified by W0 = 0 and W1 = 1, and W = 1 is specified by W1 = 0 and W0 = 1. W1 = W0 = 1 is the (all-ones) spacer and W1 = W0 = 0 is termed illegal (i.e., indeterminate) since the coding scheme must be complete [34] and unordered [35] to guarantee the delay-insensitivity.

**Table 2:** Double-rail data encoding following RTO handshaking.

Single-rail input	Double-rail encoded input		State Interpretation
W	W1	W0	
0	1	0	Binary 0
1	0	1	Binary 1
–	1	1	Spacer
–	0	0	Indeterminate

The application of inputs to a QDI circuit adhering to four-phase RTO handshaking follows the sequence of *spacer-data-spacer-data* and so forth. The spacer and data are supplied alternately implying that an RTO phase occurs between successive applications of data. The RTO phase also paves the way for a robust handshake between the current stage and next stage registers.

The four-phase RTO handshaking also involves four steps. First, Ackip = 1, and the double-rail data bus initially assumes the spacer. After the current stage register transmits the spacer, rising signal transitions would occur on all the rails of the double-rail data bus. Second, the next stage register would receive the spacer sent and would drive Ackop to 1. Third, the current stage register would wait for Ackip to assume 0 and after this occurs, it would transmit the data through the double-rail data bus. Fourth, after a finite and positive unbounded time duration elapses, the next stage register would drive Ackop to 0 and Ackip would assume 1. With this, one data transaction is said to be completed and the QDI circuit is allowed to resume the next data transaction.

In a QDI circuit, the time taken to process the data via the critical data path shown using the red dashed line in Fig 1 is called *forward latency*. The time taken to process the spacer via the critical data path is called *reverse latency*. The *cycle time* denotes the sum of forward latency and reverse latency. Importantly, the cycle time of a QDI circuit is synonymous with the clock period of a synchronous circuit. The cycle time basically determines the speed at which fresh data can be input to a QDI circuit.

The gate-level details of example completion detectors pertaining to RTZ and RTO handshaking are shown within the dotted boxes at the bottom of Fig 1. The completion detector acknowledges i.e., indicates the receipt of all the primary inputs supplied to a QDI circuit. For RTZ handshaking, 2-input OR gates are used to combine the respective rails of each double-rail encoded primary input and the outputs of all the 2-input OR gates are synchronized using a C-element/a tree of C-elements to generate Ackop. For RTO handshaking, 2-input AND gates are used instead of 2-input OR gates to combine the respective rails of each double-rail encoded primary input and the outputs of all the 2-input AND gates are synchronized using a C-element/a tree of C-elements to generate Ackop.

### 2.3. Categories of QDI circuits

**Fig 2:**
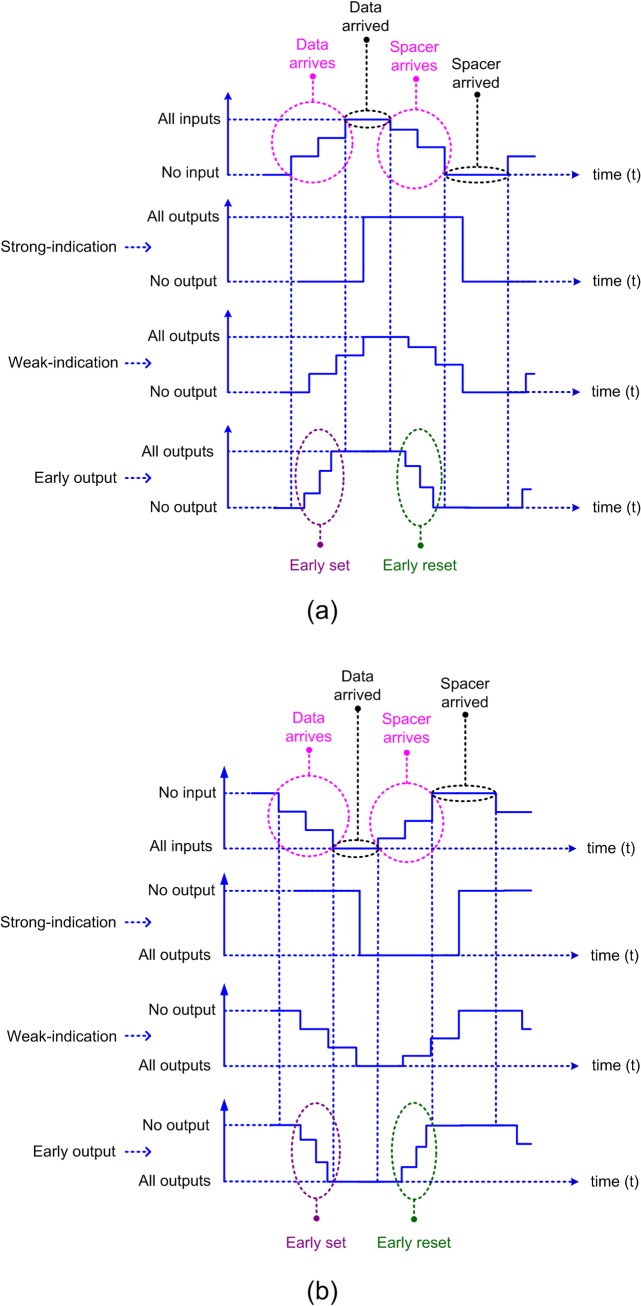
Input-cum-output timing characteristics of different categories of QDI circuits pertaining to: (a) RTZ handshaking; and (b) RTO handshaking.

QDI circuits are categorized into three types as strong-indication, weak-indication and early output. Their respective input-cum-output timing characteristics are illustrated by the representative timing diagrams shown in Fig 2.

Strong-indication circuits [37] will commence the processing only after receiving all the primary inputs (data/spacer) and then would produce the required primary outputs (data/spacer respectively). Hence, strong-indication circuits follow a strict timing regime. Weak-indication circuits are able to commence the processing after receiving some of the primary inputs (data/spacer) and after processing are able to produce all but one of the primary outputs (data/spacer respectively). However, only after receiving the last primary input will a weak-indication circuit process and produce the last primary output. Thus, weak-indication circuits are relatively relaxed in timing compared to strong-indication circuits.

The last category of QDI circuits viz. early output circuits [38] are more relaxed in timing compared to strong- and weak-indication circuits. This is because early output circuits are able to start the processing after receiving a subset of the primary inputs (data/spacer) and are able to produce all the primary outputs (data/spacer respectively) after processing. Moreover, there are two sub-categories of early output circuits, viz. the early set type and the early reset type. When an early output circuit produces the data primary output early, it is said to be of early set type. On the other hand, when an early output circuit produces the spacer primary output early, it is said to be of early reset type. The early set and reset timing behaviors of early output circuits are captured within the dotted violet and green ovals in Fig 2.

### 2.4. Characteristics of QDI circuits

Generally, early output QDI circuits are preferable to strong- and weak-indication circuits as the former can enable better optimizations in the design metrics compared to the latter. This is confirmed by the efficient designs of QDI early output adders, which are reported in the literature [39,40]. After the receipt of some primary inputs, early output circuits can process and produce all the primary outputs. This implies that the late primary input(s) may not be acknowledged by an early output circuit, which might result in wire orphans. Wire orphans and gate orphans are two issues which must be carefully addressed during the physical realization of a QDI circuit [41].

Wire orphans are not problematic as they relate to the primary inputs and they are overcome through the assumption of isochronic forks imposed on the primary input nodes. This is because the primary inputs are supplied to a QDI circuit as well as its input-side completion detector, as seen in Fig 1. All the primary inputs supplied to a QDI circuit are acknowledged by the completion detector even though some of the primary inputs may not have been acknowledged by the QDI circuit.

On the contrary, gate orphans, which are non-acknowledged signal transitions occurring on the intermediate gate outputs, pose a problem as they are likely to affect the robustness of a QDI circuit and they must be avoided. Sophisticated timing assumptions may be necessary to overcome gate orphans which may not be practically realizable [38]. Gate and wire orphans have been clearly illustrated through some examples in [32,39,42], and an interested reader may refer to these for the details.

While synthesizing a QDI circuit, large fan-in gates which are not physically realizable using a standard cell library may have to be decomposed. In such a scenario, conventional logic factoring methods such as X-factoring or quick-factoring or good factoring or set theory based factoring [43] and subsequent logic decomposition may not be suitable as they could give rise to gate orphans. Rather, a safe QDI logic factoring and decomposition [44] are necessary to eliminate the problem of gate orphans. The essential guidelines for performing safe QDI logic decomposition are discussed in [45].

Further, the monotonic cover constraint [22] should be incorporated in a QDI logic expression. For example, in the case of a sum-of-products expression, imposing the monotonic cover constraint would imply that each product term becomes mutually orthogonal to every other product term in the expression i.e., the logical conjunction of any two product terms would yield 0. This can be accomplished by transforming a sum-of-products expression into a disjoint sum-of-products expression [46]. When a QDI logic function is specified in the disjoint sum-of-products form, only one product term would become activated for the application of an input data and this would inherently satisfy the monotonic cover constraint. In other words, one signal path would be activated from a primary input to a primary output subsequent to the application of data. As a result, the switching activity and dynamic power of different implementations of a QDI circuit would be approximately the same and their total power would not vary much for different kinds of implementations.

The monotonic cover constraint facilitates the propagation of monotonic signal transitions throughout the entire depth of a QDI circuit [47]. For RTZ handshaking, rising signal transitions (0 to 1) will be encountered for the application of data, and falling signal transitions (1 to 0) will be encountered for the application of the spacer. For RTO handshaking, rising signal transitions will be encountered for the application of the spacer, and falling signal transitions will be encountered for the application of data. The monotonic cover constraint has been described through an example in [39] and an interested reader is referred to the same for the details.

## 3. Proposed QDI early output array multiplier

An N×N array multiplier requires N^2^ partial product generators to generate N^2^ partial products and N×(N–1) processing elements implemented in N levels of logic based on the carry-save adder architecture [1] with a final carry-propagate adder stage. The processing elements include full adders and partial product generators or include full adders and half adders and partial product generators. In this article, we propose a new early output partial product generator and a new weak-indication half adder for realizing efficient QDI array multipliers corresponding to RTZ and RTO handshaking.

**Fig 3:**
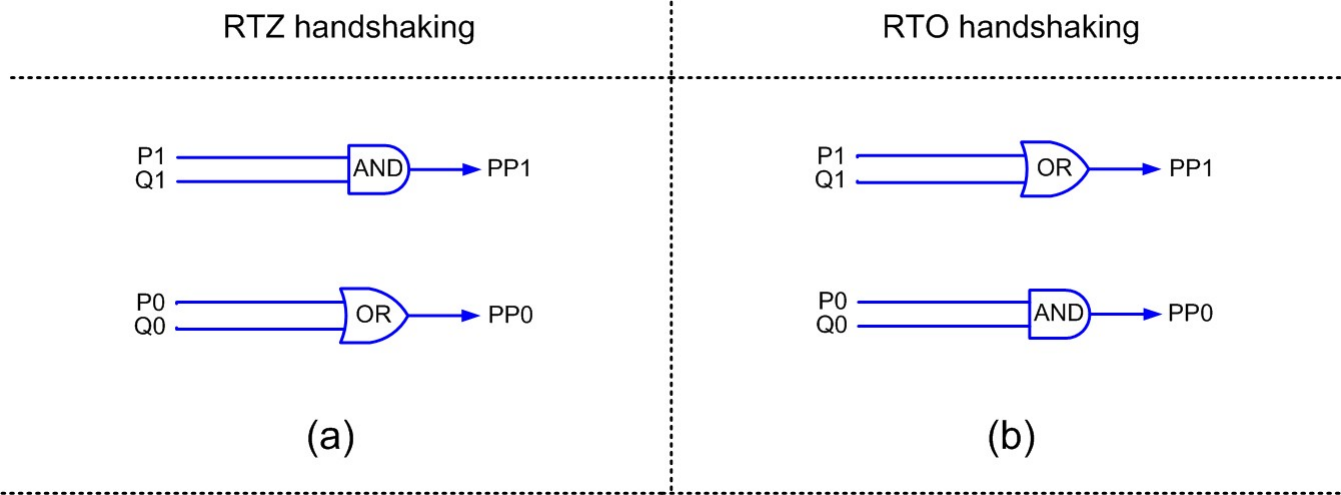
Proposed partial product generator corresponding to: (a) RTZ handshaking, and (b) RTO handshaking.

Fig 3 shows the proposed partial product generator with Fig 3A corresponding to RTZ handshaking and Fig 3B corresponding to RTO handshaking. (P1, P0) and (Q1, Q0) represent the inputs and (PP1, PP0) represent the outputs of the partial product generators.

Although Fig 3A and Fig 3B are early output types like Fig 5A and Fig 5B of [16] respectively, however, the latter require 18 transistors for a static CMOS implementation while the former require just 12 transistors for physical realization. Thus, the proposed partial product generator requires 78.6% less transistors compared to the partial product generator of [15] and 33.3% less transistors compared to the partial product generator of [16].

**Fig 4:**
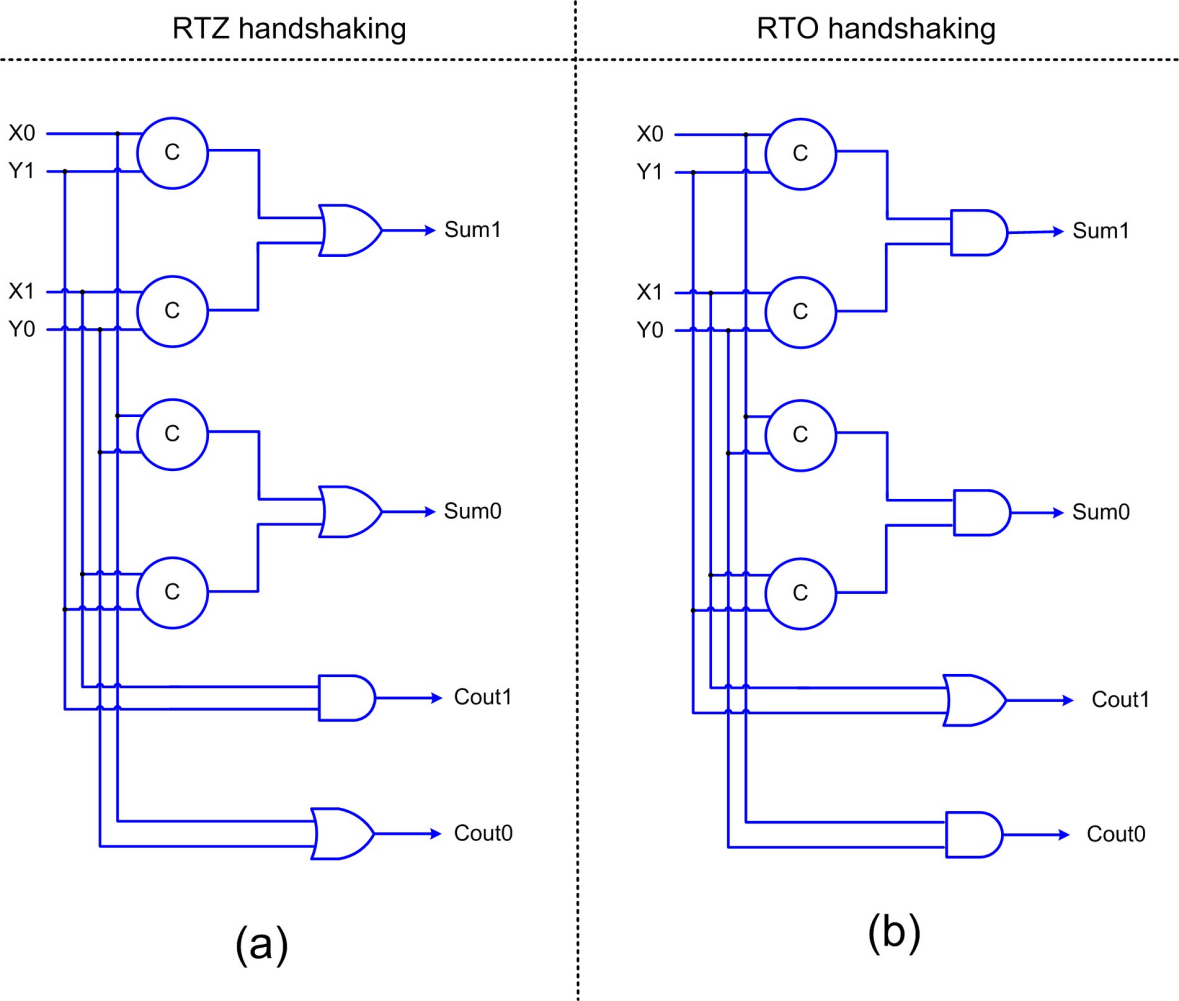
Proposed weak-indication half adder with respect to: (a) RTZ handshaking and (b) RTO handshaking.

A new weak-indication half adder is also proposed in this article, which is shown in Fig 4. The half adder basically adds two inputs (X and Y) and produces the sum (Sum) and carry (Cout) outputs. The inputs and outputs of the half adder are double-rail encoded as shown in Fig 4. Fig 4A shows the half adder design for RTZ handshaking while Fig 4B shows the half adder design for RTO handshaking. Excepting the C-elements, the duals of the gates in an asynchronous circuit pertaining to RTZ handshaking are used to obtain an asynchronous circuit that corresponds to RTO handshaking. The rules for transforming an asynchronous circuit pertaining to RTZ handshaking into one that corresponds to RTO handshaking and vice-versa are given in [48]. The proofs by induction for logic transformation between RTZ and RTO handshaking are described in [49]. In the half adders shown in Fig 4, the sum output is responsible for acknowledging the arrival of all the primary inputs while the carry output is freed from the indication. Thus, the proposed half adder corresponds to biased weak-indication [50] since the carry output logic is relaxed compared to the sum output.

**Fig 5:**
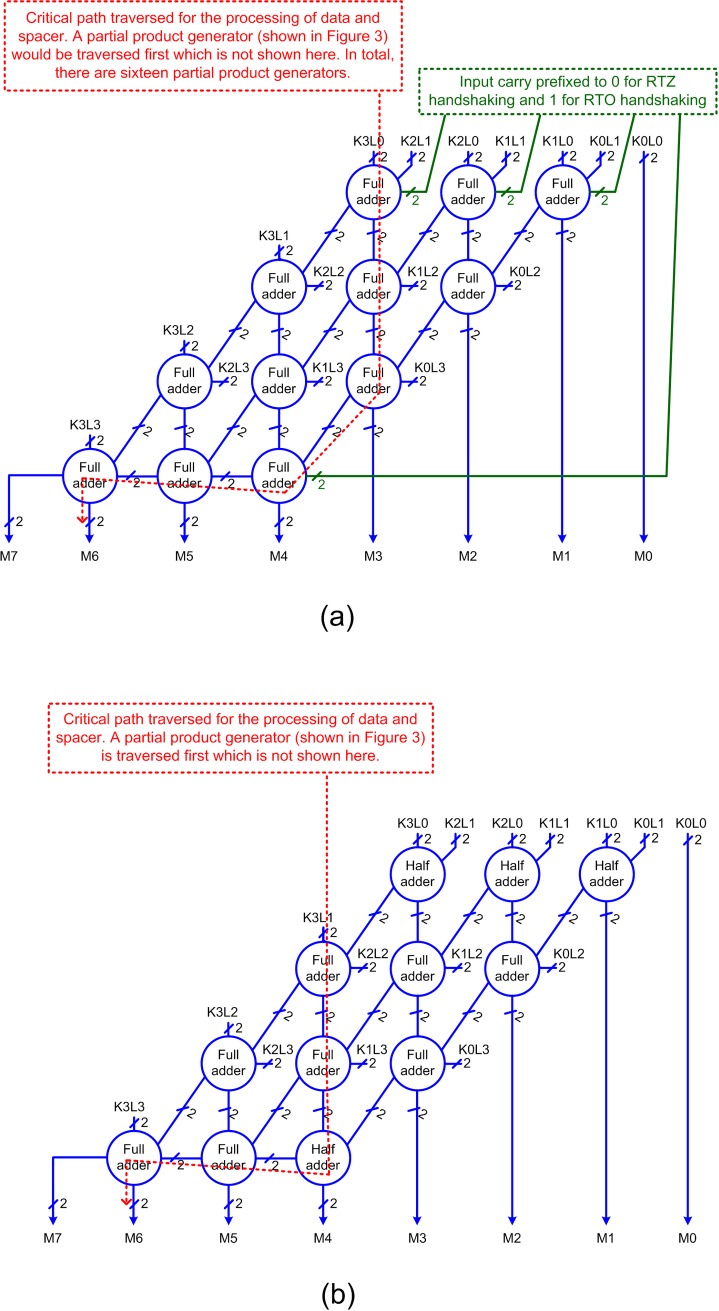
Array multiplier architecture involving: (a) full adders and partial product generators, and (b) half and full adders and partial product generators. The partial product generators are not shown in Fig 5 for brevity.

Fig 5 portrays an example 4×4 array multiplier. There are two ways of realizing a QDI array multiplier–one using full adders and partial product generators as shown in Fig 5A, and the other using half adders and full adders and partial product generators as shown in Fig 5B. In [15,16], the architecture shown in Fig 5A was used. The green lines in Fig 5A signify that the carry inputs to the respective full adders are set to 0 for RTZ handshaking and 1 for RTO handshaking. Fig 5B can be obtained from Fig 5A by eliminating the green lines in Fig 5A and replacing the full adders connected with green input lines using the half adders shown in Fig 4. For this work, we used the architecture shown in Fig 5B. In Fig 5A and 5B, K3 to K0 and L3 to L0 represent the input operands and M7 to M0 represent the outputs of the multiplier. The inputs and outputs of the multiplier are double-rail encoded. The sixteen partial product generators used to produce the partial products, signified by the generic notation ‘KjLq’ in Fig 5A and 5B, are realized using partial product generators. Note that ‘j’ and ‘q’ in ‘AjBq’ represent the numerals.

The critical paths traversed in the QDI array multipliers for the processing of data and spacer are highlighted using the red dotted lines in Fig 5A and 5B. For our optimized QDI array multiplier design, we used the proposed partial product generator shown in Fig 3A and 3B, the proposed half adder shown in Fig 4A and 4B, and the full adder presented in [33].

**Fig 6:**
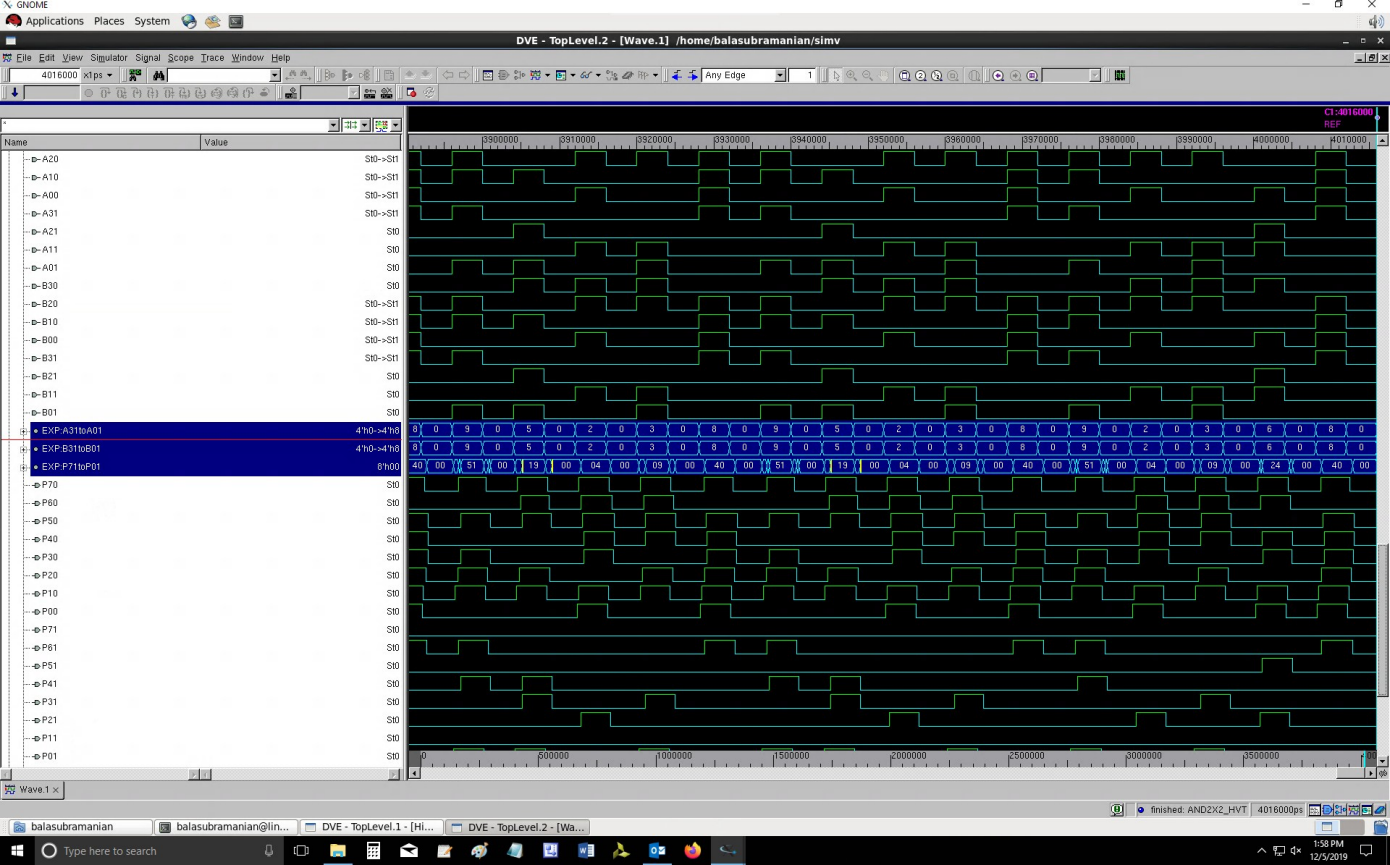
Screenshot of a portion of the simulation waveforms corresponding to the optimized QDI array multiplier design, corresponding to RTZ handshaking. The multiplier’s input and output values are displayed in hexadecimal.

**Fig 7:**
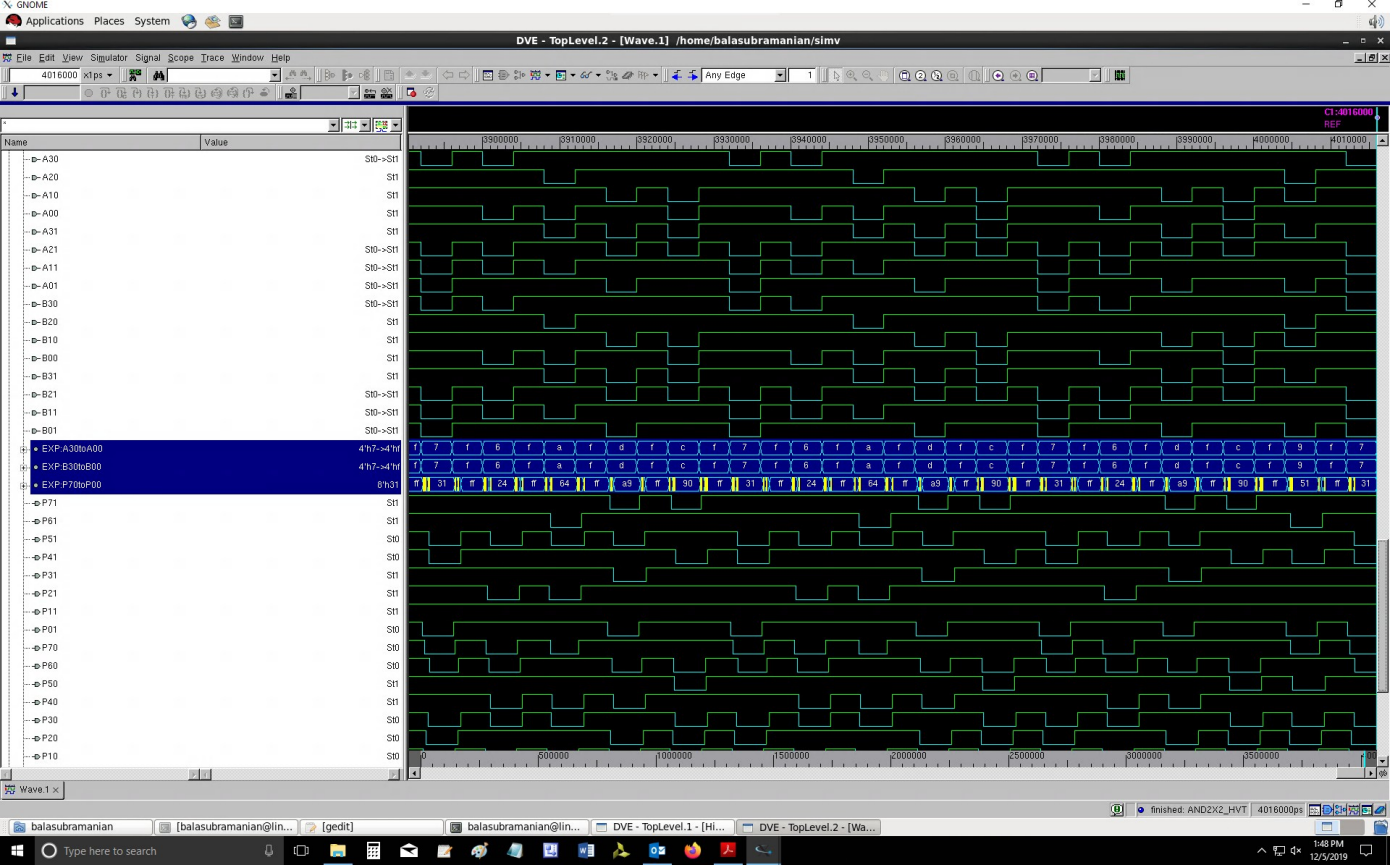
Screenshot of a portion of the simulation waveforms corresponding to the optimized QDI array multiplier design, corresponding to RTO handshaking. The multiplier’s input and output values are displayed in hexadecimal.

Screenshots of portions of simulation waveforms of the proposed QDI array multiplier designs are given in Figs 6 and 7, which correspond to RTZ and RTO handshaking respectively. The simulations were performed using Synopsys VCS tool. In Figs 6 and 7, the double-rail encoded inputs of the QDI array multiplier are represented by (A31, A30), (A21, A20), (A11, A10), (A01, A00) and (B31, B30), (B21, B20), (B11, B10), (B01, B00); the double-rail encoded outputs are represented by (P71, P70), (P61, P60), (P51, P50), (P41, P40), (P31, P30), (P21, P20), (P11, P10) and (P01, P00). In Figs 6 and 7, two input signal buses and an output signal bus are highlighted in blue for a quick reference. The inputs and output values of the QDI array multiplier displayed in Figs 6 and 7 are in hexadecimal.

In Fig 6, for RTZ handshaking, the true rails of the double-rail encoded inputs are compressed into input buses labeled ‘A31toA01’ and ‘B31toB01’. The true rails of the double-rail encoded output are compressed into an output bus labeled ‘P71toP01’. In Fig 7, for RTO handshaking, the false rails of the double-rail encoded inputs are compressed into input buses labeled ‘A30toA00’ and ‘B30toB00’. The false rails of the double-rail encoded output are compressed into an output bus labeled ‘P70toP00’. In Fig 6, it may be noted that a product ‘00h’ occurs between successive products–this is because of the RTZ which results from the application of the all-zeroes spacer subsequent to the application of an input data in the case of RTZ handshaking. In Fig 7, it may be noted that a product ‘FFh’ occurs between successive products–this is due to the RTO which results from the application of the all-ones spacer subsequent to the application of an input data in the case of RTO handshaking.

In Fig 5A, the critical path is traversed through the partial product generator (not shown in the figure), the sum logic of two full adders, the carry output logic of three full adders and finally the sum logic of the last full adder producing M6. In Fig 5B, the critical path is traversed through the partial product generator (not shown in the figure), the sum logic of two full adders, the carry output logic of a half adder and a full adder and finally the sum logic of the last full adder producing M6. Given the traversal of a smaller number of adders in Fig 5B compared to Fig 5A, the former is likely to result in lesser latencies and cycle time compared to the latter which is confirmed by the simulation results presented in the next section.

When an early output partial product generator (as shown in Fig 3A or Fig 3B), an indicating half adder (as shown in Fig 4A or Fig 4B) and an indicating full adder (as given in [33]) are used to construct a QDI array multiplier, the outputs of the partial product generators would be acknowledged by the sum outputs of the half adders and full adders in the array multiplier. Hence, the issue of gate orphans does not arise, and the multiplier is QDI.

On the contrary, supposing early output half adders and full adders are used in a QDI multiplier array, the issue of gate orphan might arise. This is because, for example, considering RTZ handshaking, an early output full adder [51] may be reset in an early fashion without having to wait to receive all the spacer inputs. Thus, it is likely that a late application of the spacer input to an early output full adder may not be acknowledged by its sum and carry outputs in the multiplier array in which case the problem of gate orphan would arise. Given this, the naïve use of early output full adders and half adders in the multiplier array is not recommended as it would affect its robustness and the asynchronous array multiplier will not be QDI. If early output half adders and full adders are to be used along with an early output partial product generator, an internal completion detector should be included whose output should be synchronized with an output bit of the multiplier to ensure that the design remains QDI.

**Fig 8:**
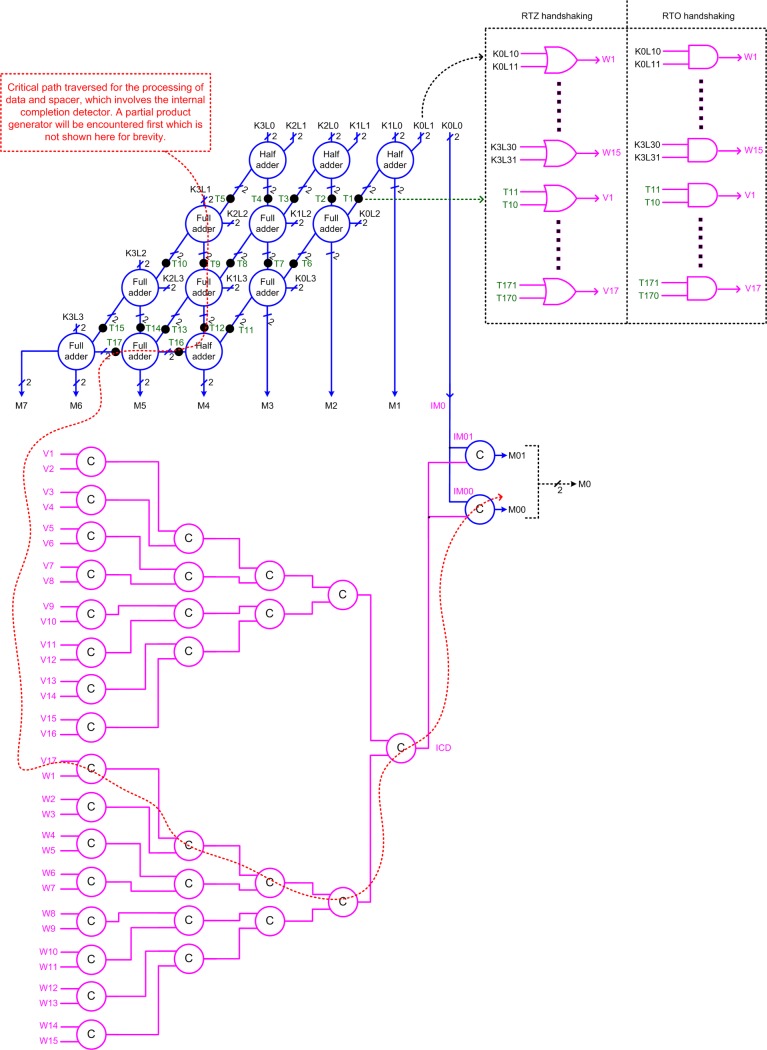
Early output QDI array multiplier comprising only early output logic blocks and an internal completion detector to ensure gate-orphan freedom.

**Fig 9:**
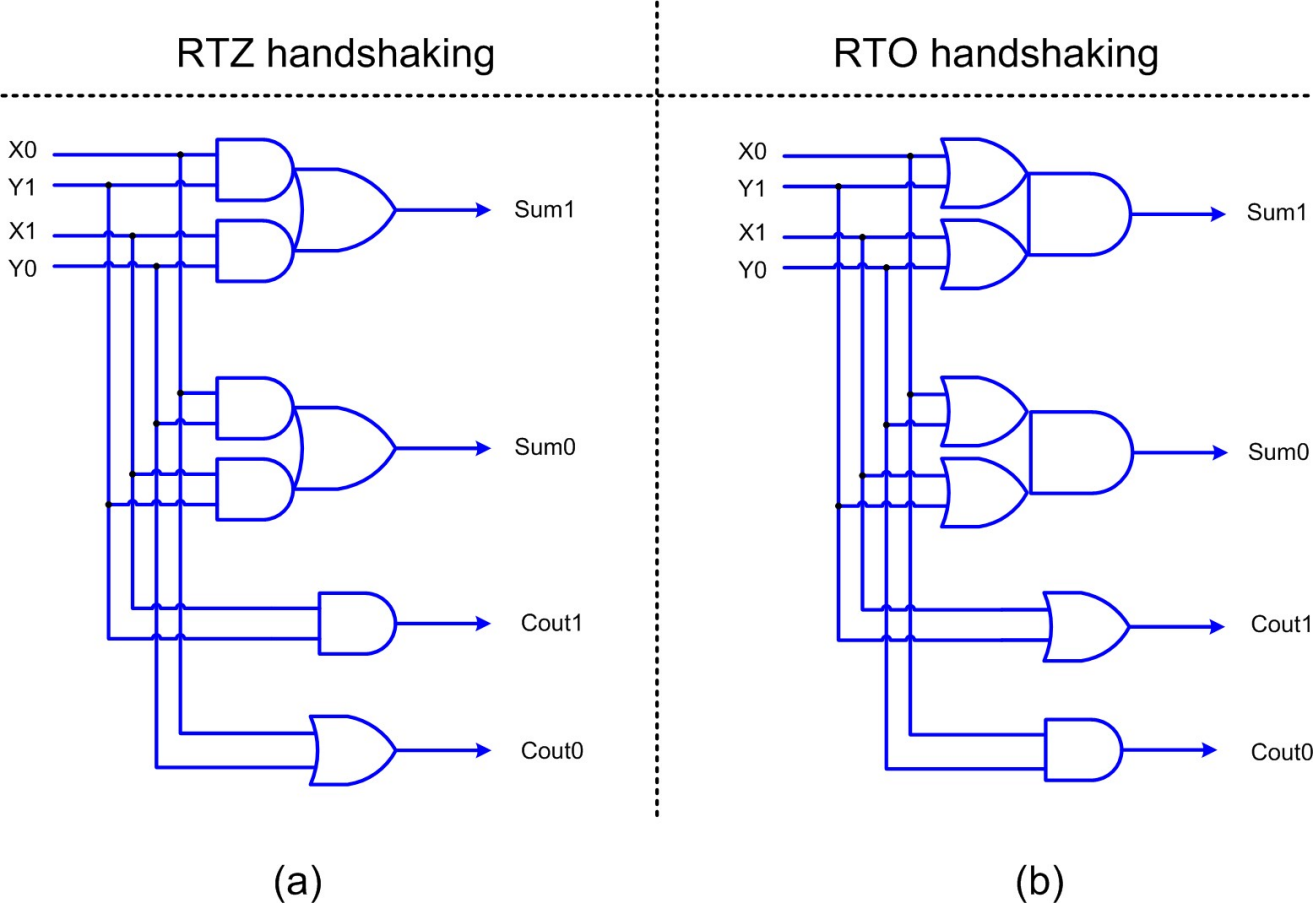
Early output half adder pertaining to: (a) RTZ handshaking; (b) RTO handshaking.

The topology of a QDI array multiplier featuring only early output building blocks such as early output half adders and full adders and an early output partial product generator is shown in Fig 8. The early output half adder is shown in Fig 9, and the early output full adder is shown in [51]. We wish to mention here that early output QDI array multipliers featuring only early output building blocks were not considered for implementation and analysis in our previous works viz. [15] and [16] other than just a mere discussion of the problem of gate orphan that is likely to arise with such an implementation.

In Figs 5 and 8, there are 16 partial products viz. K0L0, K0L1, K0L2, K0L3, K1L0, K1L1, K1L2, K1L3, K2L0, K2L1, K2L2, K2L3, K3L0, K3L1, K3L2 and K3L3 which are double-rail encoded. Of these, K0L0 corresponds to the least significant product bit of the multiplier (M0 in the case of Fig 5A and 5B, and IM0 in the case of Fig 8). Hence, excepting K0L0, the double-rails of the remaining partial products are combined using 2-input OR gates for RTZ handshaking and 2-input AND gates for RTO handshaking whose outputs are represented by W1 to W15 in Fig 8 (see the top of the right-side). T1 to T17 represents the internal outputs, highlighted by the big black dots in Fig 8, which are also double-rail encoded. Like W1 to W15, the double-rails of T1 to T17 are combined using 2-input OR gates for RTZ handshaking and 2-input AND gates for RTO handshaking whose outputs are represented by V1 to V17 in Fig 8 (see the top of the right-side of Fig 8). The use of 2-input OR gates/AND gates forms the first step in constructing an internal completion detector, as shown in Fig 1. The outputs of all the 2-input OR gates (in the case of RTZ handshaking) and 2-input AND gates (in the case of RTO handshaking) are synchronized using a C-element tree, as shown at the bottom of Fig 8. The output of the internal completion detector (ICD) is synchronized with the double-rails of the least significant product bit IM0 (i.e., IM00 and IM01) to yield the actual least significant product bit M0 (i.e., M00 and M01). However, logically, (IM01, IM00) is equivalent to (M1, M0).

The critical path traversed for the processing of data and spacer in Fig 8 is highlighted using the red dotted line. The critical path traverses through a partial product generator, the sum logic of two full adders, the carry output logic of a half adder, the carry output logic of a full adder, and six 2-input C-elements in the internal completion detector. Comparing the critical data paths of Fig 8 with Fig 5A and 5B, it is expected that the forward latency, the reverse latency and the cycle time of the former would be greater compared to the latter, which is confirmed by the simulation results given in Section 4.

To make a straightforward comparison with the early output QDI array multiplier whose architecture is portrayed by Fig 5B, we considered realizing Fig 8 using the proposed partial product generator (i.e., Fig 3A and 3B), the early output full adder of [51], and the early output half adder depicted by Fig 9. Fig 9A shows an early output half adder pertaining to RTZ handshaking and Fig 9B is its counterpart pertaining to RTO handshaking, which are derived from the early output full adder of [51]. The inputs and outputs of Fig 9 are maintained the same as Fig 4.

## 4. Results

QDI array multipliers corresponding to the architectures shown in Fig 5B and Fig 8 were physically realized based on RTZ and RTO handshaking using a 32/28 nm CMOS process [26]. QDI array multipliers corresponding to the architecture shown in Fig 5A were realized in our previous works [15] and [16], and they are referred here for the comparison. A typical case process specification with a supply voltage of 1.05 V and an operating junction temperature of 25°C was considered for the simulations. Approximately 2000 random input vectors, with half representing the data and the remaining representing the spacer, were considered as a test bench. The test bench was supplied to the multipliers assuming a cycle time of 8 ns with a 50% duty cycle (i.e., a latency of 4ns) to perform the functional simulations, as done in [15,16], to pave the way for a direct comparison post-simulation. There is a logical equivalence between the input vectors comprising the test benches which pertain to RTZ and RTO handshaking. The switching activity captured through the functional simulations was used to estimate the average (total) power dissipation. Synopsys tools were used to estimate the design parameters viz. cycle time, area, and average power dissipation, and these are given in Table 3. The simulation set-up was maintained the same as in [15,16] to pave the way for a legitimate comparison.

For the ease of referencing, we use certain legends in Table 1 to refer to the different QDI array multipliers. Z1 and Z2 in Table 3 are early output QDI array multipliers which correspond to RTZ handshaking. O1 and O2 are their early output counterpart designs which correspond to RTO handshaking. Z1 and O1 are QDI array multipliers corresponding to the architecture shown in Fig 8, which are realized entirely using early output building blocks viz. the early output half adders shown in Fig 9A and 9B, our early output full adder of [51], and the proposed early output partial product generators shown in Fig 3A and 3B. On the other hand, Z2 and O2 correspond to the architecture shown in Fig 5B, which utilize the proposed early output partial product generators shown in Fig 3A and 3B, the proposed weak-indication half adders shown in Fig 4A and 4B, and ur early output full adder of [51].

**Table 3:** Design parameters of newly realized QDI array multipliers pertaining to RTZ and RTO handshaking, based on implementation using a 32/28 nm CMOS technology.

**Handshaking Protocol**	**Multiplier Legend**	**Cycle Time (ns)**	**Area (μm**^**2**^**)**	**Power (μW)**
RTZ	Z1	3.82	594.95	1051
	Z2	3.04	579.45	1020
RTO	O1	3.78	594.95	1050
	O2	2.98	579.45	1019

The forward latency of a QDI circuit is the same as the critical path delay of a synchronous circuit, which is directly estimated through static timing analysis. The reverse latency is however estimated using the timing information of the gates obtained from the gate-level timing analysis as done in [15,16,39,40]. It is seen from Table 3 that Z1 and O1 require the same area, and Z2 and O2 also require the same area despite the differences in the handshake schemes. This is because some of the dual gates in the cell library [26] have the same area. For examples, the minimum size 2-input AND and OR gates in [26] have the same area of 2.03μm^2^, and the minimum size AO22 and OA22 gates in [26] have the same area of 2.54μm^2^. Nevertheless, the power dissipation components and propagation delays of these gate duals differ.

Two general inferences can be derived by comparing Table 3 of this work with Table 1 of [16]: (i) RTO handshaking typically leads to slightly better optimizations (i.e., reductions) in the design metrics compared to RTZ handshaking, and (ii) compared to the weak indication QDI array multipliers, the early output QDI array multipliers report less cycle time, occupy less silicon area, and dissipate less power. Generally, the early output timing model leads to enhanced optimizations of the design metrics compared to the other timing models, which was found to be the case with adders [39,40] and multipliers [16]. This is because the early output timing model is more relaxed compared to the other timing models and this is mainly because conventional gates are used more often than the C-elements.

It is worth noting here that the early output QDI array multipliers of [16] report better optimized design metrics than the weak-indication QDI array multipliers of [15]. Hence, we consider ZM7 (EO) of [16], which represents the optimized design in the existing literature with respect to RTZ handshaking, for comparison with Z1 and Z2 of this work. Likewise, we consider OM7 (EO) of [16], which represents the optimized design in the existing literature with respect to RTO handshaking, for comparison with O1 and O2 of this work. The comparison between ZM7 (EO) of [16] and Z1 of this work shows that the former has a 4.2% less cycle time but the latter requires 14% less area and dissipates 12.7% lesser power. The comparison between OM7 (EO) of [16] and O1 of this work shows that the former has a 9.5% less cycle time but the latter requires 14% less area and dissipates 12.6% lesser power.

However, it is noticed from Table 3 that the QDI array multipliers Z2 and O2 comprising the proposed early output partial product generator and the proposed weak-indication half adder report reduced cycle time, less area occupancy, and minimized power dissipation compared to Z1 and O1 with respect to RTZ and RTO handshaking respectively.

Compared to Z1 (O1), which corresponds to the architecture shown in Fig 8, the proposed Z2 (O2), which corresponds to the architecture shown in Fig 5B, reports a 20.4% (21.1%) reduction in cycle time without any area or power penalty. The reduction in cycle time for the proposed designs Z2 and O2 is attributed to the fewer logic elements encountered in the critical path. In the case of Z1 or O1, the critical path traverses through an internal completion detector which is not the case with Z2 or O2 as they do not require an internal completion detection. The early output full adder of [51], used in Z1/O1, requires 33.3% less area than the weak-indication full adder of [33] used in Z2/O2. Also, the early output half adder shown in Fig 9, used for Z1/O1, requires 57% less area than the weak-indication half adder shown in Fig 4 that is used for Z2/O2. Further, the same early output partial product generators shown in Fig 3A and 3B have been used to realize Z1/O1 and Z2/O2 respectively. As a result, it may appear that Z2/O2 would consume more silicon than Z1/O1, which is not true though. This is because despite the compact early output building blocks used, Z1/O1 eventually ends up consuming slightly more area than Z2/O2, as seen from Table 3. This is mainly due to the internal completion detector included in Z1/O1, which is absent in Z2/O2. Due to the lesser area and the non-use of an internal completion detector, which would experience regular switching activity for the application of data and spacer, Z2/O2 is found to dissipate less average power compared to Z1/O1. Hence, the proposed Z2 and O2 outperform Z1 and O1 respectively in terms of all the design metrics.

Compared to ZM7 (EO) of [16], the proposed Z2 reports a 17% reduction in cycle time, a 16.1% reduction in area, and a 15.3% reduction in power dissipation. Compared to OM7 (EO) of [16], the proposed O2 reports a 13% reduction in cycle time, a 16.1% reduction in area, and a 15.2% reduction in power dissipation. Hence, the proposed QDI array multipliers are speed, area, and power efficient compared to the existing designs in the literature. Comparing Z2 and O2, it is observed that O2 has a slightly reduced cycle time by 2% without incurring any area or power penalty. This implies that the RTO handshaking is preferable to RTZ handshaking for realizing QDI array multipliers.

With respect to a synchronous digital circuit, the power-delay product (PDP) [52] serves as a qualitative figure-of-merit for quantifying its low power/energy efficiency. PDP is the product of average power dissipation and the critical path delay. In the case of a QDI asynchronous circuit, the power-cycle time product (PCTP) serves as the equivalent figure-of-merit for quantifying the low power/energy efficiency. PCTP is calculated as the product of average power dissipation and the cycle time. The cycle time represents the speed of a QDI circuit because it determines the rate at which fresh data can be input to the circuit. Power dissipation and cycle time are desired to be less in a QDI circuit, which implies that the PCTP is also desired to be less. Thus, the lesser the PCTP, the better the power/energy efficiency of a QDI circuit.

The PCTPs of the QDI array multipliers discussed in this work and those given in [15,16] were computed by multiplying their corresponding power and cycle time. To normalize the PCTPs, the highest PCTP pertaining to a handshake protocol was considered as the baseline, and this was used to divide the PCTPs of all the QDI array multipliers pertaining to that handshake protocol. Hence, the minimum value of PCTP signifies an optimum QDI array multiplier with respect to a specific handshake scheme. Based on the calculations, it is found that the proposed Z2 achieves a 29.6% reduction in PCTP compared to ZM7 (EO) of [16], and the proposed O2 achieves a 26.1% reduction in PCTP compared to OM7 (EO) of [16]. Further, Z2 achieves a 22.8% reduction in PCTP compared to Z1, and O2 achieves a 23.5% reduction in PCTP compared to O1. Moreover, O2 has a slightly reduced PCTP than Z2 by 2% implying that the former corresponding to RTO handshaking is slightly more energy efficient than the latter which corresponds to RTZ handshaking.

## 5. Conclusions

Multiplication is a widely used and important arithmetic operation which is realized using a multiplier. This paper presented speed, power, area, and energy-efficient asynchronous QDI array multipliers pertaining to RTZ and RTO handshaking. To achieve this, novel designs of an asynchronous partial product generator and an asynchronous half adder were presented in this paper. The proposed designs of the early output partial product generator and the weak-indication half adder were used in conjunction with our weak-indication full adder of [33] to realize an optimum QDI array multiplier design. It is also discussed how the proposed QDI array multipliers are notably better than the existing QDI array multipliers and also better than the QDI array multipliers realized entirely using early output building blocks.
